# Acute beriberi neuropathy mimicking Guillain-Barré syndrome after a strict vegetarian diet

**Published:** 2017-04-04

**Authors:** Anis Riahi, Malek Mansour, Ines Bedoui, Hajer Derbali, Mariem Messelmani, Jamel Zaouali, Ridha Mrissa

**Affiliations:** Department of Neurology, Military Hospital, Tunis, Tunisia

**Keywords:** Thiamin, Deficiency, Beriberi, Polyneuropathies, Guillain-Barré Syndrome, Diet, Vegetarian

Vitamin B1 (thiamin) is required in the metabolism of carbohydrates, lipids, proteins, and energetic metabolism within Krebs cycle. It has also an essential role in acetylcholine synthesis and neurotransmission.^1^ Thiamin deficiency can lead to various manifestations such as Gayet-Wernicke syndrome, wet beriberi with congestive heart failure, or dry beriberi with peripheral neuropathy. We report herein a case of Guillain-Barré-like neuropathy caused by acute vitamin B1 deficiency in a young girl consecutive to a strict vegetarian diet.

A 14-year-old girl with unremarkable medical history has presented with numbness and weakness of lower limbs developing insidiously for a month. Two weeks before admission, weakness progressed to upper limbs and cramping pain in legs has appeared. The patient had difficulties at swallowing and she has become unable to walk 1 day before admission. In the preceding 3 months, the patient has undergone a strict diet made only of vegetables and yogurt to get thinner. Her weight has dropped from 66 to 42 kg. At examination, the girl was thin and pale and her voice was dysphonic. Her body mass index (BMI) was 15.24 kg/m². She was not able to walk without double help. Her muscular testing revealed distal-dominant weakness in her four limbs. Deep tendon reflexes were absent. She had also hypoesthesia in her legs. Cranial nerves examination noted a facial diparesis. Psychological interrogatory did not reveal any behavioral or alimentary disorder. Nerve conduction studies showed a severe sensory and motor neuropathy with axonal and demyelinating mechanism predominantly in lower limbs. It has revealed decreased motor potentials amplitude with conduction blocks, abnormal temporal dispersion of the motor responses, and the sensory potentials were not recordable (Table 1).

Cerebrospinal fluid analysis made on the day of admission revealed normal protein content (0.29 g/l) and no leukocytes. Thiamin plasma level was low: 13 ng/ml (normal range: 20-50 ng/ml). Concentration of vitamin B12 (cobalamin) and B9 (folic acid) were normal. Routine hematological and biochemical tests showed no abnormalities. Electrocardiogram was also normal. 

**Table 1:** Nerve conduction study

**Stimulation site**	**Median nerve**					**Tibial nerve**			**Sural nerve**	
	**Motor**			**Sensory**		**Motor**			**Sensory**	
	**MCV (m/s)**	**DL (ms)**	**CMAP (mV)**	**SCV (m/s)**	**SNAP (µV)**	**MCV (m/s)**	**DL (ms)**	**CMAP (mV)**	**SCV (m/s)**	**SNAP (µV)**
Right										
Distal	61.7	8.0	4.0	NR	NR	70.0	8.5	1.5	NR	NR
Proximal			1.3					1.0		
Left										
Distal	51.4	9.3	3.0	NR	NR	63.8	7.8	2.8	NR	NR
Proximal			1.9					1.1		

CMAP: Compound muscle action potential; DL: Distal latency; MCV: Motor nerve conduction velocity; NR: Not recordable; SCV: Sensory nerve conduction velocity; SNAP: Sensory nerve action potential

Intravenous (IV) thiamin supplementation was administered at 300 mg 2 times a day for 1 week, associated to multivitamin perfusion.

Daily physiotherapy, psychologist, and nutritionist consulting were also practiced. Subsequent follow-up showed slow but significant improvement. The girl was discharged after 2 weeks on intramuscular thiamin injections (300 mg bd) for four additional weeks; then, she was placed on oral thiamin tablets. One month later, she was able to walk by herself with dropping feet and she had no more dysphonia. Six months later on physiotherapy and balanced alimentation, her examination showed almost normal gait, normal deep tendon reflexes, and her BMI was 22.49 kg/m².

Thiamin deficiency results habitually from chronic alcoholism, hyperemesis gravidarum (HG), and gastric bypass or cancer surgeries.^1^ Its prevalence is underestimated since symptoms are often latent and some conditions such as high carbohydrates intake or excessive physic activity can make it manifest.^1^ Historical cases of dry beriberi were known since the 19^th^ century in sailors after long sea voyages and poor diet. Nowadays, alimentary thiamin shortage is rare in the developed and in most of developing country. It may happen in cases of anorexia nervosa or after long-term parenteral alimentation without vitamin supplementation.^2^^,^^3^ Our patient did not present personality disorder or distorted body self-perception indicating anorexia nervosa. Strict vegetarian diet without anorexia nervosa is an exceptional cause of vitamin B1 deficiency. In fact, the body stores in normal conditions 25-30 mg of thiamin, mostly in metabolically active organs such as the heart, kidneys, and brain. Due to its rapid turnover, thiamin stock is rapidly exhausted within 2-3 weeks in case of lack of alimentary supply.^1^ In the gastrointestinal tract, thiamin absorption is an active mechanism and the mucosal transporters are situated mostly in the duodenum. Threshold of thiamin intestinal absorption is about 10 mg/day, and it decreases in case of malnutrition.^1^ Thereby, oral thiamin supplementation is not effective in beriberi and Gayet-Wernicke syndrome.

Complications of thiamin deficiency can be life-threatening as in Gayet-Wernicke syndrome and in wet beriberi (also called Shoshin beriberi). They can also alter patient’s functional outcome as in dry beriberi and in rare cases of optic neuritis. In beriberi neuropathy, nerve conduction studies show usually sensory predominant axonal neuropathy.^2^^-^^4^ Motor symptoms are mostly latent and may be worsened by associated thiamin deficiency-induced myopathy. Acute beriberi neuropathy mimicking Guillain-Barré syndrome (GBS) is rare and must be considered face to person at risk of thiamin deficiency. Our patient’s electromyography (EMG) showed some classical finding of GBS such as the presence of partial motor conduction blocks and abnormal temporal dispersion of motor responses.^5^ Furthermore, the rapid evolution and the ascension from lower limbs to upper limbs then to face remind GBS natural course.

In the literature, similar cases of dry beriberi mimicking GBS are seldom reported.^3^ Koike, et al. found that 7 out of 11 (64%) neuropathies due to thiamin deficiency in patients who had dietary imbalance show acute progression.^3^ Nerve conduction study in the same paper revealed sensory and motor neuropathy more marked in the lower limbs and with mixed axonal and demyelinating mechanism.^3^^,^^5^

Thiamin plasma level can be assessed by high-performance liquid chromatography conveniently since the 1980’s. Nevertheless, thiamin plasma concentration analysis may delay and patient must be placed on thiamin supplementation without waiting for the result if he matches the clinical feature of vitamin B1 deficiency. As in Gayet-Wernicke syndrome, early treatment is crucial to prevent permanent complications such as residual deficits. Usually, functional recovery is achieved within the 6 months, but sensory symptoms may linger.^4^ In our patient, IV immunoglobulins were discussed at the first time, but the history of alimentary imbalance directed the diagnosis to beriberi. Fortunately, the good evolution advocated this diagnosis.

In conclusion, dry beriberi becomes rare at present days. Nevertheless, physicians must recall this diagnosis face to an acute neuropathy, especially in patients with a history of alimentary disorders. Early thiamin supplementation is mandatory for a suitable recovery.

## Notes:

**How to cite this article:** Riahi A, Mansour M, Bedoui I, Derbali H, Messelmani M, Zaouali J, et al. Acute beriberi neuropathy mimicking Guillain-Barré syndrome after a strict vegetarian diet. Iran J Neurol 2017; 16(2): 100-2.
